# 5-Hydr­oxy-8-nitro-1,4-naphthoquinone

**DOI:** 10.1107/S1600536808021594

**Published:** 2008-07-19

**Authors:** Daniel Teoh-Chuan Tan, Hasnah Osman, Azlina Harun Kamaruddin, Reza Kia, Hoong-Kun Fun

**Affiliations:** a School of Chemical Sciences, Universiti Sains Malaysia, 11800 USM, Penang, Malaysia; b School of Chemical Engineering, Universiti Sains Malaysia, Seri Ampangan, 14300 Nibong Tebal, Penang, Malaysia; c X-ray Crystallography Unit, School of Physics, Universiti Sains Malaysia, 11800 USM, Penang, Malaysia

## Abstract

The title compound, C_10_H_5_NO_5_, features an intra­molecular O—H⋯O hydrogen bond, forming a six-membered ring with an *S*(6) ring motif. The nitro group makes a dihedral angle of 71.66 (5)° with the plane of the benzene ring to which it is bound. The two rings are almost coplanar, with a dihedral angle of 4.44 (5)°. Short inter­molecular distances between the centroids of the six-membered rings [3.7188 (6)–3.8299 (6) Å] indicate the existence of π–π inter­actions. The inter­esting features of the crystal structure are the short inter­molecular O⋯O and O⋯N inter­actions. The crystal packing is stabilized by intra­molecular O—H⋯O and inter­molecular C—H⋯O (×3) hydrogen bonds, and C—H⋯π inter­actions.

## Related literature

For related literature on hydrogen-bond motifs, see Bernstein *et al.* (1995 ▶). For values of bond lengths, see Allen *et al.* (1987 ▶). For related literature, see, for example: Guingant & Barreto (1987 ▶); Larsen *et al.* (1996 ▶); Krohn *et al.* (2004 ▶); Krohn *et al.* (2004 ▶); Cui *et al.* (2006 ▶); Anuradha *et al.* (2006 ▶).
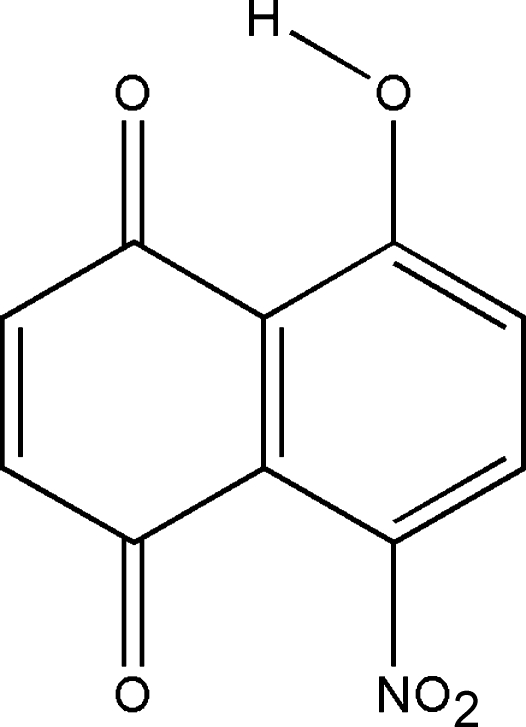


## Experimental

### 

#### Crystal data

C_10_H_5_NO_5_
*M* *_r_* = 219.15
Monoclinic, 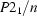

*a* = 8.6809 (2) Å
*b* = 8.4250 (2) Å
*c* = 12.1845 (3) Å
β = 93.946 (1)°
*V* = 889.02 (4) Å^3^
*Z* = 4
Mo *K*α radiation
μ = 0.14 mm^−1^
*T* = 100.0 (1) K
0.35 × 0.14 × 0.13 mm

#### Data collection

Bruker SMART APEXII CCD area-detector diffractometer
Absorption correction: multi-scan (*SADABS*; Bruker, 2005 ▶) *T*_min_ = 0.900, *T*_max_ = 0.982
22792 measured reflections
3028 independent reflections
2493 reflections with *I* > 2σ(*I*)
*R*_int_ = 0.041

#### Refinement

*R*[*F* ^2^ > 2σ(*F* ^2^)] = 0.041
*wR*(*F* ^2^) = 0.119
*S* = 1.11
3028 reflections
165 parameters
H atoms treated by a mixture of independent and constrained refinement
Δρ_max_ = 0.50 e Å^−3^
Δρ_min_ = −0.23 e Å^−3^

### 

Data collection: *APEX2* (Bruker, 2005 ▶); cell refinement: *APEX2*; data reduction: *SAINT* (Bruker, 2005 ▶); program(s) used to solve structure: *SHELXTL* (Sheldrick, 2008 ▶); program(s) used to refine structure: *SHELXTL*; molecular graphics: *SHELXTL*; software used to prepare material for publication: *SHELXTL* and *PLATON* (Spek, 2003 ▶).

## Supplementary Material

Crystal structure: contains datablocks global, I. DOI: 10.1107/S1600536808021594/at2591sup1.cif

Structure factors: contains datablocks I. DOI: 10.1107/S1600536808021594/at2591Isup2.hkl

Additional supplementary materials:  crystallographic information; 3D view; checkCIF report

## Figures and Tables

**Table 1:** Selected interatomic and centroid–centroid distances (Å) *Cg*1 and *Cg*2 are the centroids of the C1–C5/C10 and C5–C10 rings, respectively.

*Cg*1⋯*Cg*2^i^	3.7188 (6)
*Cg*1⋯*Cg*2^i^	3.8299 (6)
O2⋯O5^i^	2.9940 (11)
O5⋯O5^ii^	3.0367 (11)
O5⋯N1^ii^	3.0608 (11)

Symmetry codes: (i) 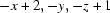; (ii) 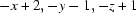.

**Table 2:** Hydrogen-bond geometry (Å, °) *Cg*1 is the centroid of the C1–C5/C10 ring.

*D*—H⋯*A*	*D*—H	H⋯*A*	*D*⋯*A*	*D*—H⋯*A*
O1—H1*O*1⋯O2	0.889 (18)	1.769 (19)	2.5695 (10)	148.5 (16)
C2—H2⋯O3^iii^	0.969 (15)	2.547 (16)	3.1853 (12)	123.4 (12)
C3—H3⋯O5^ii^	0.970 (15)	2.577 (15)	3.3827 (13)	140.6 (11)
C7—H7⋯O1^iv^	0.982 (16)	2.561 (16)	3.1851 (13)	121.4 (12)
C8—H8⋯*Cg*1^v^	0.950 (15)	2.976 (14)	3.6548 (11)	129.5 (11)

Symmetry codes: (ii) 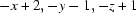; (iii) 
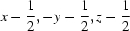
; (iv) 
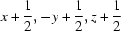
; (v) 
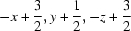
.

**Fig. 1.**
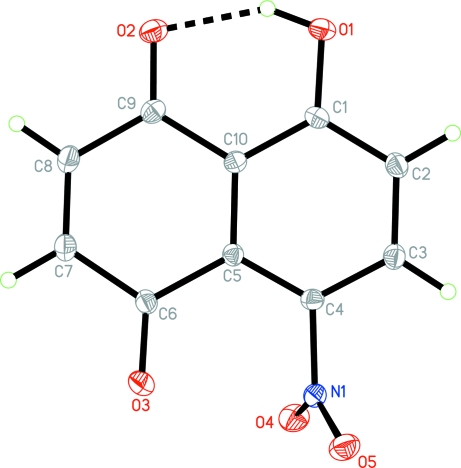
The molecular structure of the title compound, showing 50% probability displacement ellipsoids and the atomic numbering. Intramolecular hydrogen bond is drawn as a dashed line.

**Fig. 2.**
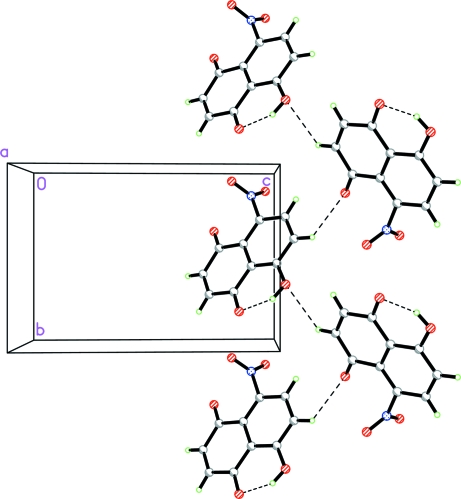
The crystal packing of (I) shows a one-dimensional infinite chain along the [010] direction when viewed down the a-axis. Intramolecular and intermolecular interactions are drawn as dashed lines.

## supplementary crystallographic information

### Comment

5-Hydroxy-1,4-naphthoquinone (juglone) and its 5-acetoxy-2-bromo analogue is the essential dienophile in the highly convergent and regiospecific Diels-Alder synthesis of ochromycinone (Guingant & Barreto, 1987; Larsen *et al.*, 1996; Krohn *et al.*, 2004) a type of natural anthraquinone which exhibits remarkable antibiotic and antitumour activities (Krohn *et al.*, 2004; Cui *et al.*, 2006). Our aim is to prepare aromatic ring substituted juglone analogues for the purpose of synthesizing new ochromycinone analogues. The title compound was prepared by the direct nitration of juglone with nickel(II) nitrate. The method outlined previously (Anuradha *et al.*, 2006) predicted a *ortho*-nitro product. However the product that we obtained is a *para*-nitro product.

Compound (I), ( Fig. 1), features an intramolecular O—H···O hydrogen bond to form a six-membered ring, producing a *S*(6) ring motif (Bernstein *et al.*, 1995). The bond lenghts and angles are within the normal ranges (Allen *et al.*, 1987). The two phenyl rings are almost coplanar with the dihedral angle of 4.44 (5)°. The nitro group is not coplanar with the benzene ring and its orientation is indicated by the dihedral angle of 71.66 (5)° with the plane of the benzene ring to which it is bound. The short intermolecular distances between the centroids of six-membered rings [3.7188 (6) - 3.8299 (6) Å] prove existence of π–π interactions (Table 1). The interesting feature of the crystal structure is the short intermolecular O···O and O···N interactions (Table 1). The crystal packing,(Fig. 2), of the compound shows one-dimensional infinite chains along the *b* axis.The crystal packing is stabilized by the intramolecular O—H···O, intermolecular C—H···O hydrogen bonds, π–π, and C—H···π interactions.

### Experimental

8-Nitro-5-hydroxy-1,4-naphthoquinone was prepared from 5-hydroxy-1,4-naphthoquinone by the protocol outlined by (Anuradha *et al.*, 2006). Single crystals of 8-nitro-5-hydroxy-1,4-naphthoquinone was obtained by slow evaporation of a chloroform solution at 286 K° C.

### Refinement

All of the H-atoms were located from the difference Fourier map and refined freely.

### Crystal data

**Table:**

C_10_H_5_NO_5_	*F*_000_ = 448
*M**_r_* = 219.15	*D*_x_ = 1.637 Mg m^−^^3^
Monoclinic, *P*2_1_/*n*	Mo *K*α radiation λ = 0.71073 Å
Hall symbol: -P 2yn	Cell parameters from 5257 reflections
*a* = 8.6809 (2) Å	θ = 2.8–31.8º
*b* = 8.4250 (2) Å	µ = 0.14 mm^−^^1^
*c* = 12.1845 (3) Å	*T* = 100.0 (1) K
β = 93.9460 (10)º	Block, brown
*V* = 889.02 (4) Å^3^	0.35 × 0.14 × 0.13 mm
*Z* = 4	

### Data collection

**Table:**

Bruker SMART APEXII CCD area-detector diffractometer	3028 independent reflections
Radiation source: fine-focus sealed tube	2493 reflections with *I* > 2σ(*I*)
Monochromator: graphite	*R*_int_ = 0.041
*T* = 100.0(1) K	θ_max_ = 31.8º
φ and ω scans	θ_min_ = 2.8º
Absorption correction: multi-scan(SADABS; Bruker, 2005)	*h* = −12→12
*T*_min_ = 0.901, *T*_max_ = 0.982	*k* = −12→12
22792 measured reflections	*l* = −18→17

### Refinement

**Table:**

Refinement on *F*^2^	Secondary atom site location: difference Fourier map
Least-squares matrix: full	Hydrogen site location: inferred from neighbouring sites
*R*[*F*^2^ > 2σ(*F*^2^)] = 0.041	H atoms treated by a mixture of independent and constrained refinement
*wR*(*F*^2^) = 0.119	*w* = 1/[σ^2^(*F*_o_^2^) + (0.0671*P*)^2^ + 0.1177*P*] where *P* = (*F*_o_^2^ + 2*F*_c_^2^)/3
*S* = 1.11	(Δ/σ)_max_ = 0.001
3028 reflections	Δρ_max_ = 0.50 e Å^−^^3^
165 parameters	Δρ_min_ = −0.22 e Å^−^^3^
Primary atom site location: structure-invariant direct methods	Extinction correction: none

### Special details

**Table:**

Experimental. The low-temperature data was collected with the Oxford Cyrosystem Cobra low-temperature attachment.
Geometry. All e.s.d.'s (except the e.s.d. in the dihedral angle between two l.s. planes) are estimated using the full covariance matrix. The cell e.s.d.'s are taken into account individually in the estimation of e.s.d.'s in distances, angles and torsion angles; correlations between e.s.d.'s in cell parameters are only used when they are defined by crystal symmetry. An approximate (isotropic) treatment of cell e.s.d.'s is used for estimating e.s.d.'s involving l.s. planes.
Refinement. Refinement of *F*^2^ against ALL reflections. The weighted *R*-factor *wR* and goodness of fit *S* are based on *F*^2^, conventional *R*-factors *R* are based on *F*, with *F* set to zero for negative *F*^2^. The threshold expression of *F*^2^ > σ(*F*^2^) is used only for calculating *R*-factors(gt) *etc*. and is not relevant to the choice of reflections for refinement. *R*-factors based on *F*^2^ are statistically about twice as large as those based on *F*, and *R*- factors based on ALL data will be even larger.

### Fractional atomic coordinates and isotropic or equivalent isotropic displacement parameters (Å^2^)

**Table:**

	*x*	*y*	*z*	*U*_iso_*/*U*_eq_	
O1	0.60929 (9)	0.14448 (9)	0.47684 (6)	0.01684 (17)	
O2	0.70729 (9)	0.29533 (9)	0.65117 (6)	0.01718 (17)	
O3	1.13635 (9)	−0.14728 (9)	0.74975 (6)	0.01826 (17)	
O4	0.97394 (10)	−0.42986 (9)	0.67891 (6)	0.02146 (19)	
O5	1.11804 (9)	−0.37894 (9)	0.54429 (6)	0.01912 (18)	
N1	1.01058 (10)	−0.34927 (10)	0.60140 (7)	0.01427 (18)	
C1	0.71200 (11)	0.03181 (12)	0.50785 (8)	0.01280 (18)	
C2	0.71889 (11)	−0.10405 (12)	0.44177 (8)	0.01457 (19)	
C3	0.81830 (11)	−0.22605 (12)	0.47321 (8)	0.01431 (19)	
C4	0.91370 (11)	−0.21081 (11)	0.56968 (8)	0.01219 (18)	
C5	0.91680 (11)	−0.07504 (11)	0.63367 (7)	0.01156 (18)	
C6	1.03288 (11)	−0.05166 (12)	0.72840 (8)	0.01326 (19)	
C7	1.02414 (12)	0.09636 (13)	0.79222 (8)	0.0172 (2)	
C8	0.91880 (12)	0.20847 (12)	0.76649 (8)	0.0168 (2)	
C9	0.80517 (11)	0.19086 (12)	0.67159 (8)	0.01387 (19)	
C10	0.81225 (11)	0.04769 (11)	0.60319 (8)	0.01189 (18)	
H8	0.9139 (16)	0.3051 (18)	0.8063 (12)	0.022 (4)*	
H2	0.6513 (17)	−0.1106 (19)	0.3752 (12)	0.025 (4)*	
H3	0.8222 (16)	−0.3231 (18)	0.4307 (12)	0.020 (3)*	
H7	1.1034 (18)	0.1097 (19)	0.8530 (13)	0.029 (4)*	
H1O1	0.620 (2)	0.223 (2)	0.5255 (16)	0.049 (5)*	

### Atomic displacement parameters (Å^2^)

**Table:**

	*U*^11^	*U*^22^	*U*^33^	*U*^12^	*U*^13^	*U*^23^
O1	0.0161 (3)	0.0151 (4)	0.0187 (4)	0.0031 (3)	−0.0031 (3)	0.0017 (3)
O2	0.0197 (4)	0.0144 (4)	0.0176 (3)	0.0040 (3)	0.0029 (3)	0.0005 (3)
O3	0.0190 (4)	0.0170 (4)	0.0179 (3)	0.0025 (3)	−0.0051 (3)	0.0005 (3)
O4	0.0285 (4)	0.0150 (4)	0.0210 (4)	0.0010 (3)	0.0023 (3)	0.0054 (3)
O5	0.0161 (3)	0.0181 (4)	0.0234 (4)	0.0026 (3)	0.0032 (3)	−0.0025 (3)
N1	0.0159 (4)	0.0110 (4)	0.0155 (4)	−0.0001 (3)	−0.0015 (3)	−0.0010 (3)
C1	0.0114 (4)	0.0132 (4)	0.0136 (4)	0.0000 (3)	−0.0003 (3)	0.0021 (3)
C2	0.0142 (4)	0.0156 (5)	0.0135 (4)	−0.0011 (3)	−0.0018 (3)	−0.0004 (3)
C3	0.0152 (4)	0.0138 (4)	0.0138 (4)	−0.0011 (3)	0.0000 (3)	−0.0021 (3)
C4	0.0122 (4)	0.0110 (4)	0.0133 (4)	0.0007 (3)	0.0005 (3)	0.0008 (3)
C5	0.0120 (4)	0.0112 (4)	0.0114 (4)	−0.0010 (3)	0.0002 (3)	−0.0001 (3)
C6	0.0143 (4)	0.0130 (4)	0.0122 (4)	−0.0011 (3)	−0.0012 (3)	0.0005 (3)
C7	0.0203 (5)	0.0157 (5)	0.0150 (4)	−0.0008 (4)	−0.0030 (3)	−0.0026 (4)
C8	0.0204 (5)	0.0144 (5)	0.0153 (4)	−0.0006 (4)	−0.0005 (3)	−0.0035 (3)
C9	0.0155 (4)	0.0123 (4)	0.0141 (4)	−0.0002 (3)	0.0031 (3)	0.0002 (3)
C10	0.0123 (4)	0.0110 (4)	0.0124 (4)	0.0001 (3)	0.0011 (3)	0.0004 (3)

### Geometric parameters (Å, °)

**Table:**

O1—C1	1.3388 (11)	C3—C4	1.3964 (13)
O1—H1O1	0.89 (2)	C3—H3	0.970 (15)
O2—C9	1.2367 (12)	C4—C5	1.3836 (13)
O3—C6	1.2211 (12)	C5—C10	1.4087 (13)
O4—N1	1.2229 (11)	C5—C6	1.4921 (13)
O5—N1	1.2274 (11)	C6—C7	1.4744 (14)
N1—C4	1.4741 (12)	C7—C8	1.3369 (15)
C1—C2	1.4031 (14)	C7—H7	0.982 (16)
C1—C10	1.4091 (13)	C8—C9	1.4748 (14)
C2—C3	1.3793 (14)	C8—H8	0.950 (15)
C2—H2	0.969 (15)	C9—C10	1.4699 (13)
			
Cg1···Cg2^i^	3.7188 (6)	O5···O5^ii^	3.0367 (11)
Cg1···Cg2^i^	3.8299 (6)	O5···N1^ii^	3.0608 (11)
O2···O5^i^	2.9940 (11)		
			
C1—O1—H1O1	107.7 (12)	C4—C5—C6	122.07 (8)
O4—N1—O5	124.96 (9)	C10—C5—C6	119.72 (8)
O4—N1—C4	117.90 (8)	O3—C6—C7	120.63 (9)
O5—N1—C4	117.04 (8)	O3—C6—C5	121.69 (9)
O1—C1—C2	118.06 (8)	C7—C6—C5	117.58 (8)
O1—C1—C10	121.79 (9)	C8—C7—C6	122.22 (9)
C2—C1—C10	120.15 (9)	C8—C7—H7	121.9 (10)
C3—C2—C1	119.94 (9)	C6—C7—H7	115.8 (9)
C3—C2—H2	121.4 (9)	C7—C8—C9	121.51 (9)
C1—C2—H2	118.6 (9)	C7—C8—H8	122.7 (9)
C2—C3—C4	119.26 (9)	C9—C8—H8	115.8 (9)
C2—C3—H3	121.6 (9)	O2—C9—C10	121.66 (9)
C4—C3—H3	119.1 (9)	O2—C9—C8	119.96 (9)
C5—C4—C3	122.53 (9)	C10—C9—C8	118.38 (9)
C5—C4—N1	121.19 (8)	C5—C10—C1	119.86 (9)
C3—C4—N1	116.27 (8)	C5—C10—C9	120.27 (8)
C4—C5—C10	118.10 (8)	C1—C10—C9	119.86 (9)
			
O1—C1—C2—C3	−177.19 (9)	O3—C6—C7—C8	175.40 (10)
C10—C1—C2—C3	3.41 (15)	C5—C6—C7—C8	−1.17 (15)
C1—C2—C3—C4	−1.45 (15)	C6—C7—C8—C9	−0.45 (16)
C2—C3—C4—C5	−2.37 (15)	C7—C8—C9—O2	178.62 (10)
C2—C3—C4—N1	176.71 (9)	C7—C8—C9—C10	−1.51 (15)
O4—N1—C4—C5	72.88 (12)	C4—C5—C10—C1	−2.00 (14)
O5—N1—C4—C5	−110.47 (10)	C6—C5—C10—C1	174.21 (8)
O4—N1—C4—C3	−106.21 (10)	C4—C5—C10—C9	176.79 (8)
O5—N1—C4—C3	70.44 (11)	C6—C5—C10—C9	−7.00 (14)
C3—C4—C5—C10	4.07 (14)	O1—C1—C10—C5	178.97 (9)
N1—C4—C5—C10	−174.97 (8)	C2—C1—C10—C5	−1.65 (14)
C3—C4—C5—C6	−172.05 (9)	O1—C1—C10—C9	0.17 (14)
N1—C4—C5—C6	8.92 (14)	C2—C1—C10—C9	179.56 (9)
C4—C5—C6—O3	4.45 (15)	O2—C9—C10—C5	−174.82 (9)
C10—C5—C6—O3	−171.60 (9)	C8—C9—C10—C5	5.31 (14)
C4—C5—C6—C7	−179.01 (9)	O2—C9—C10—C1	3.97 (15)
C10—C5—C6—C7	4.94 (13)	C8—C9—C10—C1	−175.90 (9)

Symmetry codes: (i) −*x*+2, −*y*, −*z*+1; (ii) −*x*+2, −*y*−1, −*z*+1.

### Hydrogen-bond geometry (Å, °)

**Table:**

*D*—H···*A*	*D*—H	H···*A*	*D*···*A*	*D*—H···*A*
O1—H1O1···O2	0.889 (18)	1.769 (19)	2.5695 (10)	148.5 (16)
C2—H2···O3^iii^	0.969 (15)	2.547 (16)	3.1853 (12)	123.4 (12)
C3—H3···O5^ii^	0.970 (15)	2.577 (15)	3.3827 (13)	140.6 (11)
C7—H7···O1^iv^	0.982 (16)	2.561 (16)	3.1851 (13)	121.4 (12)
C8—H8···Cg1^v^	0.950 (15)	2.976 (14)	3.6548 (11)	129.5 (11)

Symmetry codes: (iii) *x*−1/2, −*y*−1/2, *z*−1/2; (ii) −*x*+2, −*y*−1, −*z*+1; (iv) *x*+1/2, −*y*+1/2, *z*+1/2; (v) −*x*+3/2, *y*+1/2, −*z*+3/2.
